# The Effect of a Nutritional Intervention with the Use of Biofortified Beef Meat on Selected Biochemical Parameters in Blood from Older Adults

**DOI:** 10.3390/nu16142281

**Published:** 2024-07-16

**Authors:** Lisia Bertonha Correa, Janaina Silveira da Silva, Marcus Antonio Zanetti, Nara Regina Brandão Cônsolo, Karina Pfrimer, Arlindo Saran Netto

**Affiliations:** 1 Department of Animal Science, College of Animal Science and Food Engineering, University of Sao Paulo, Pirassununga 13635-900, Brazil; lisiabc@yahoo.com.br (L.B.C.); zanettimarcusa@gmail.com (M.A.Z.); 2 Department of Animal Nutrition and Production, College of Veterinary Medicine and Animal Science, University of Sao Paulo, Pirassununga 13635-900, Brazil; nara.consolo@usp.br; 3 Department of Biotechnology and Nutrition, University of Ribeirão Preto (UNAERP), Ribeirao Preto 14096-900, Brazil; kpfrimer@unaerp.br; 4 Program of Post-Graduation Nutrition and Metabolism, Department of Health Sciences, School Medical of Ribeirão Preto, University of Sao Paulo, Avenida Bandeirantes, 3900, Ribeirão Preto 14049-900, Brazil

**Keywords:** canola oil, cholesterol, selenium, vitamin E

## Abstract

This study aimed to investigate the effects of meat biofortified with antioxidants and canola oil on the health of older adults through blood parameters. Eighty institutionalized older persons were divided into four groups who received the following treatments: C-control meat with 46 µg/kg of meat with selenium, 3.80 g/kg of meat with vitamin E and 0.78 g/100 g of meat with conjugated linoleic acid (CLA); A-antioxidant meat with 422 µg/kg of meat with selenium, 7.65 g/kg of meat with vitamin E and 0.85 g/100 g of meat with CLA; O-oil meat with 57 µg/kg of meat with selenium, 3.98 g/kg of meat with vitamin E and 1.27 g/100 g of meat with CLA; OA-oil and antioxidant meat with 367 µg/kg of meat with selenium, 7.78 g/kg of meat with vitamin E and 1.08 g/100 g of meat with CLA. Blood samples were collected at 0, 45 and 90 days after the start of meat intake. Older adults who consumed ANT (A and AO) meat had higher concentrations of selenium (*p* = 0.039), vitamin E and HDL (higher concentrations of high-density lipoprotein, *p* = 0.048) in their blood. This study demonstrates that the consumption of Se- and vitamin E-biofortified meat increases the concentration of these metabolites in blood from older adults.

## 1. Introduction

In the past few years, several studies have shown that older adults can suffer from malnutrition, due to their inability to utilize most of the nutrients from their diet. This impaired nutritional status in older adults is frequently associated with falls and bone fractures, higher oxidative stress and weaker antioxidant defense systems [1,2,3]. Additionally, improving eating behavior can minimize the risk of the prevalence of many degenerative and chronic diseases [4,5] such as diabetes [6], atherosclerosis [7], hypertension [1], Alzheimer’s disease and dementia [8].

Beef represents an important constituent of the human diet and is one of the major sources of protein in many countries. Beyond amino acids, beef also provides an abundance of fatty acids, vitamins and minerals with obvious nutritional benefits to human health. For this reason, the beef industry is currently undergoing changes towards fortifying produced beef, which can be achieved by supplementing the diets of animals with so-called “good fat” (*n*-3, *n*-6 and *n*-9) and antioxidants to enhance its concentration on meat, making it nutritionally healthier for human consumption [9,10,11].

Canola is an important source of long-chain polyunsaturated fatty acids (PUFAs), and feeding ruminants with this oilseed is related to changes in meat fatty acid profile toward a high level of *n*-3 polyunsaturated fatty acid [11]. In sequence, this compound is associated with a decrease in human heart disease, inflammation processes, improvement in brain development and the immune system [12,13]. Selenium is an essential trace mineral for both humans and animals. It is an important component required for the synthesis of dozens of selenoproteins, which have various biological functions involved in redox homeostasis, immunity, reproduction, thyroid hormone metabolism and cognitive processes [14].

In humans, severe selenium deficiency can be associated with low immunity, increased risks of cancer, bone diseases, several chronic diseases and loss of cognitive functions [15]. Similarly, vitamin E also induces the synthesis of some important compounds involved in the antioxidant defense process of the body. The nutritional benefits of vitamin E are often related to anti-cancer activity [16], inhibit cardiovascular and bone diseases [17] and inhibit neurodegenerative disorders [18].

Therefore, it is increasingly known that so-called good fat, Se and vitamin E have many potential health benefits beyond meeting basic nutritional requirements. Besides that, many older people are experiencing malnutrition [19], and nutrient requirements may be even higher in frail older adults who are at risk of malnutrition because of acute or chronic illness [20], which could influence their quality of life.

Thus, the hypothesis is that the enrichment of meat with PUFAs and antioxidants can be a way to improve the nutritional quality of the final product (meat), which brings benefits to meat consumers. Therefore, this study aims to investigate the effects of meat biofortified with antioxidants (selenium and vitamin E) and canola oil on the health of older adults through selected biochemical blood parameters.

## 2. Materials and Methods

This study is part of a larger experiment that has been already published by Correa et al. [11,21]. The first stage of this experiment was to produce meat biofortified with antioxidants and oil, and then the second stage was to feed older adults this meat. Both studies were approved by the Animals Ethics Committee (CEUA nº 14.1.1455.74.0.) and the Human Research Ethics Committee (nº 1683/2011 and clinical trials, 1038/2006).

### 2.1. Producing Biofortified Meat

Briefly, an experiment with forty-eight Nellore bulls was performed at the Animal Science and Food Engineering College of Pirassununga-SP, Brazil. The animals were randomly distributed in 2 × 2 factorial arrangement (12/treatment) with two levels of oil in the diet (no inclusion and 3% canola oil) and two levels of antioxidants in the diet (no inclusion and 2.5 mg of Se/kg of dry matter (DM) + 500 UI of vitamin E/kg of DM); C: basal diet, A: basal diet + 2.5 mg Se/kg and 500 UI vitamin E/kg, O: basal diet + 3% canola oil, AO: basal diet + 3% canola oil + 2.5 mg Se/kg and 500 UI vitamin E/kg.

Animals were fed for 12 weeks, and at 24 h after slaughter, steaks from the longissimus thoracis muscle at the 12th rib level were packed and conditioned at 2.0 ± 1.0 °C to supply to humans later.

### 2.2. Subjects

All participants and their family members were contacted in presentations made in the São Vicente de Paula, in Leme, SP, Brazil, when the purposes and methods of the study were presented and residents were invited to participate in this study.

A total of 80 institutionalized older adult individuals were recruited, between 50 and 70 years old, male and female, who resided in a controlled environment where meals were provided centrally. There were no changes to the volunteers’ diets; they already received meat as part of their diet. Even so, the diet was monitored daily by a nutritionist. Besides that, regular assessments and check-ins with participants helped monitor compliance with study protocols. Any significant deviations or lifestyle changes were documented and addressed accordingly.

For the assurances of lifestyle stability, the subjects were educated about the importance of maintaining their usual lifestyle habits throughout the study duration, and detailed protocols were established and communicated to subjects and caregivers to ensure adherence to study requirements. The volunteers were physically examined to check overall health status, including vital signs (blood pressure, heart rate, respiratory rate), general appearance and ability to eat meat (without restrictions). Besides that, they were tested for biochemical and lipid profile measure cholesterol levels (total cholesterol, HDL, LDL) to assess cardiovascular risk factors and blood glucose.

All volunteers who participated in the experiment were aware of the study through a lecture that was previously held, and they signed the consent form approved by the Ethics Committee, and they were not allowed to eat other foods than those served.

At the institute, there was a nutritionist responsible for the formulation of the older adults’ diet and supervising the preparation of all meals. Older adults were randomly assigned to four treatments according to the meat enriched through the animals’ diets as follows: C = control meat, A = meat biofortified with antioxidants (selenium and vitamin E), O = meat biofortified with canola oil, AO = meat biofortified with antioxidants (selenium and vitamin E) plus canola oil. The nutritional composition of biofortified raw meat produced by Nellore bulls fed diets with antioxidants (selenium and vitamin E) and canola oil is shown in Table 1. More details about the meat quality can be found at Correa et al. [11,21].

The older adults received bracelets of different colors according to the treatment they were in. All meals offered to the older adults had the same procedure (three different ways: ground, steak and pot meat), being prepared in specific pans and planned to present similar nutrient contents. This study was not a double-blinded study. Two daily meals of 150 g of meat each were prepared for each individual in their respective treatment and offered three times a week for fourteen weeks. The biofortified meat was given to the older subjects two times a day, at 11:00 h for lunch (150 g of meat) and at 16:00 h for dinner (150 g of meat), to compare the treatments and to verify how biofortified meat can help individuals to reach the recommended daily intake values of these nutrients.

### 2.3. Data Collection and Chemical Analysis

Blood samples were collected at baseline (day 0, before receiving the treatments), 45 and 90 days, in the morning, after a 12 h fast. For the serum total cholesterol, high-density lipoprotein (HDL), low-density lipoprotein (LDL), triglycerides and glucose analyses, La-borlab commercial kits (Laborlab^®^, Guarulhos, São Paulo, Brazil) were used with a Jaffe^®^ automatic analyzer.

Selenium in serum was analyzed by the fluorimetric method by Olson et al. [22] and improved by Wetter and Ullrey [23]. The content of vitamin E was determined by reversed-phase high-performance liquid chromatography using a fluorescence detector with the methods of Liu et al. [24].

### 2.4. Statistical Analysis

All statistical analyses were conducted using SAS version 9.4 for Windows (SAS Institute Inc., Cary, NC, USA). Data were analyzed as a 2 × 2 factorial scheme in a completely randomized design using the MIXED procedure with the following treatments: C = control meat with 46 µg/kg of meat with selenium, 3.80 g/kg of meat with vitamin E and 0.78 g/100 g of meat with conjugated linoleic acid (CLA); A = antioxidant meat with 422 µg/kg of meat with selenium, 7.65 g/kg of meat with vitamin E and 0.85 g/100 g of meat with CLA; O = oil meat with 57 µg/kg of meat with selenium, 3.98 g/kg of meat with vitamin E and 1.27 g/100 g of meat with CLA; AO = oil and antioxidant meat with 367 µg/kg of meat with selenium, 7.78 g/kg of meat with vitamin E and 1.08 g/100 g of meat with CLA.

The model included the fixed effects of antioxidants (ANT, A and AO), oil (OIL, C and O) and their interaction (OIL*ANT). Also, the time of blood collection and its interaction with diet (Diet*Time) were taken into account in the model.

Firstly, the normality of residuals and the homogeneity of variances were verified using the UNIVARIATE procedure. Each person was considered as an experimental unit following the model:

Yijk = µ + ANTi + OILj + (OIL × ANT)ij + eijk

where Yijk = dependent variable; µ = the general population mean; i = effect of antioxidants in the diet (ANT); j = effect of oil in the diet and eijk = the unexplained residual element assumed to be independent and normally distributed.

The means were obtained through LSMEANS, and the significance level was ≤5%.

## 3. Results

### 3.1. Blood Serum Selenium and Vitamin E

There was no oil*antioxidant (O*A) interaction (*p* > 0.05) for Se and vitamin E concentrations in the blood from older adults (Table 2). There was a time effect: the concentrations of selenium (*p* < 0.001) increased after 90 days of biofortified meat consumption, while vitamin E concentrations (*p* < 0.001) decreased.

**Figure 1:**
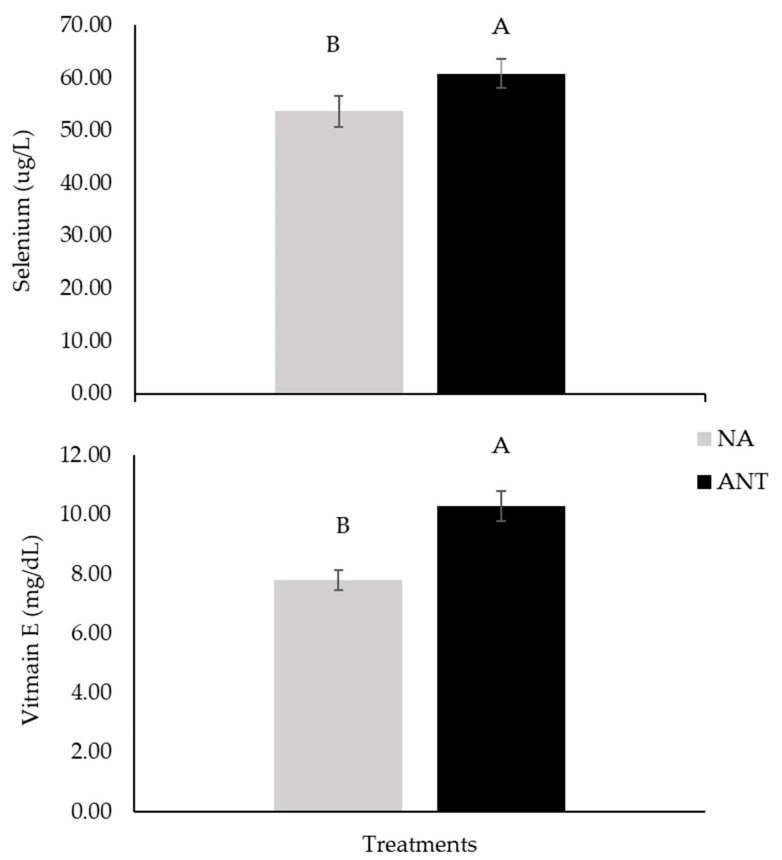
Serum selenium and vitamin E concentrations in older adults, after 90 days of supplementation with biofortified meat. Data are means ± standard error (*n* = 40). NA = no antioxidants (composed of C + O), and ANT = antioxidants (composed of A + AO). Different capital letters denote statistical significance (*p* < 0.05) among group that received antioxidants (ANT) and one other that did not receive antioxidants (NA).

There was a main effect of ANT (antioxidant effect): the participants who consumed the biofortified meat with antioxidants (A and AO) had 17% more selenium (*p* = 0.039, Figure 1) and 10% more vitamin E (*p* = 0.0425) in the blood than those who consumed NA (no antioxidants, groups C and O).

### 3.2. Blood Serum Biochemical Analysis

There was a time effect: concentrations of total cholesterol (*p* < 0.001), HDL (*p* < 0.001), glucose (*p* = 0.0167) and LDL + VLDL (*p* = 0.0023) decreased after 90 days of consumption. However, concentrations of triglycerides (*p* = 0.0039) increased (Table 3).

**Figure 2:**
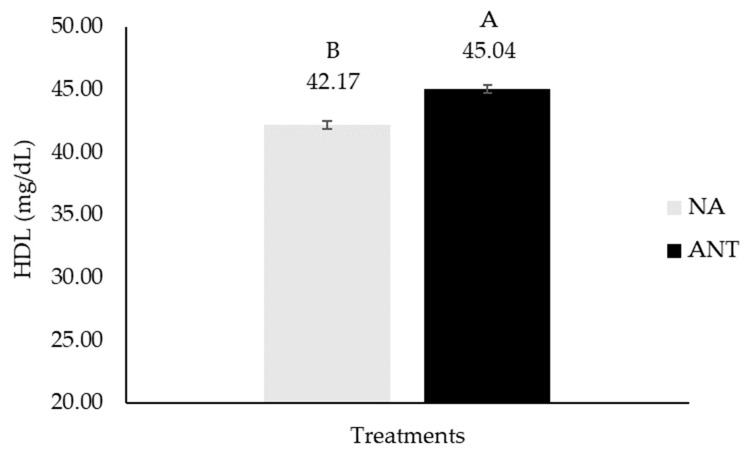
HDL concentrations in older adults, after 90 days of supplementation with biofortified meat. Data are means ± standard error (*n* = 40). NA = no antioxidants (composed of C + O), and ANT = antioxidants (composed of A + AO). Different capital letters denote statistical significance (*p* < 0.05) among group that received antioxidants (ANT) and one other that did not receive antioxidants (NA).

There was a main effect of antioxidants (ANT): the older adults who consumed the biofortified meat with antioxidants (A and AO) had 6.8% more HDL (*p* = 0.048, Figure 2) than those who consumed NA (no antioxidants, groups C and O).

## 4. Discussion

Aging is an inevitable process characterized by changes in health and physiology which can lead to the development of a high risk of nutritional deficiencies that are either due to low dietary intake, impairment in the mechanism of absorption or failure to convert to active forms. This situation affects cognitive functioning and can lead to the development of degenerative diseases [25]. This impaired nutritional status in older adults is frequently associated with falls and bone fractures, higher oxidative stress and weaker antioxidant defense systems [1,2]. Thus, improving eating behavior is fundamental to minimizing the risk of prevalence of many degenerative and chronic diseases [4] such as diabetes [6], atherosclerosis [7], hypertension [2] Alzheimer’s disease and dementia [8].

Due to older adults failing to meet their requirements for nutrients through dietary sources due to their inadequate food intake, the use of dietary supplements has been in great demand [25]. Beef is an important natural source of nutrients, and the fortification of livestock feeds is a strategy shown to successfully boost nutrient levels even further [26,27].

Several studies have investigated the association between animal nutrition and the quality of their products like meat [11], milk [28,29] and eggs [30] to meet the demand for consumers’ concern with healthy living [31]. In this context, the importance and power of some nutrients or compounds is already known to improve human health and decrease cases of some diseases, especially those correlated with chronic inflammation, degenerative processes and consumers’ welfare [24].

The antioxidants selenium and vitamin E are interrelated in that cells require both for complete protection. Selenium is an important micronutrient that, as an antioxidant, prevents the formation of free radicals, protecting the body from oxidative aggression through a series of selenoproteins, mainly glutathione peroxidase (GPx) and selenoprotein P [32]. Globally, Se deficiency is more prevalent than Se poisoning [33], and Se deficiency can result in a decrease in human immunity and can threaten human health [34].

Animal products are an important source of proteins, vitamins, minerals and fatty acids for human health and consumed daily by humans. An improvement in those products’ nutritional quality can be made by changes in animal diets [11,35]. In this research, older adults who ingested biofortified meat, produced by beef cattle fed vitamin E and selenium, showed greater blood levels of those antioxidants and HDL cholesterol fractions as compared to the older adults who ingested meat from cattle without antioxidant supplementation.

According to the WHO and FAO (2001), the recommended (IR) Se and vitamin E intake is 55 μg of Se/day and 15 mg of vitamin E/day for adults. If we make an estimate, 150 g of biofortified meat (with 0.42 mg Se/kg meat and 7.65 mg vitamin E/kg) would provide, approximately, 63 μg of Se (100% of Se IR) and 1.15 mg of vitamin E (7.7% of Se IR). In our study, the increase in selenium and vitamin E in the blood of older adults proves that both were present in biofortified meat and were absorbed to the point of altering their serum levels in humans. This confirms what Wu et al. [36] said about the importance of producing Se-biofortified meat products from animals fed Se-enriched diets and concludes that this approach could be essential for higher dietary Se intake.

In other words, providing biofortified meat to older adults is an efficient way to raise or maintain the levels of these two antioxidants in the human body, which can prevent many aging-related diseases. Indeed, aging is a complex phenomenon that plays a significant role in the nutritional status, health and brain capacity integrity of older adults. Aging is closely related to oxidative stress and it can be accelerated by the increasing levels of reactive oxygen species (ROS); when antioxidative defense is not well stimulated, oxidative stress can lead to lipid and protein (enzyme) oxidation and cellular and DNA damages, resulting in cases of chronic inflammation, which has been correlated with several diseases in elderly people [37].

Unfortunately, we did not evaluate reactive oxygen species (ROS) parameters, so more investigation with a greater number of experimental units and a longer period of experimentation can be interesting to better explain the effect of meat biofortified with antioxidants.

Another important point is that cardiovascular diseases remain, globally, the leading cause of mortality, with hypercholesterolemia as the major risk factor. Thus, many studies have been searching for nutraceutical and phytochemical drugs with the ability to lower cholesterol and that have minimal side effects. Among them, selenium is on the list due to its heart function and the relationship between blood selenium level and lipid profiles documented in recent articles [38,39].

In the present study, we did not find a significant effect of treatments on total cholesterol, but it was observed that HDL cholesterol levels are positively associated with the antioxidants group, as previously described by others’ works [40,41,42,43]. In longitudinal analyses with elderly volunteers, the increasing plasma selenium concentrations were associated with decreasing total cholesterol levels and increasing HDL cholesterol levels [44]. Similarly, a China cross-sectional study with 1859 participants (aged 65 or older) observed that higher selenium levels were significantly associated with a lower risk of low-HDL [42]. In the USA, selenium in adults also was associated with increasing HDL [41].

Meat products have been rejected by many consumers due to bad publicity in terms of the adverse effects of animal fats on human health; the fat contains a low proportion of unsaturated fatty acids (UFA), which are correlated with the incidence of cardiovascular disease and cancer [45,46]. Experiments using canola oil in the diet of animals during the fattening period observed an improvement in the meat quality and may increase the amount of intramuscular fat with a healthier fatty acid profile (oleic acid, CLA and eicosapentaenoic acid) [46] while reducing the lipid oxidative substances in meat [47]. Based on the literature presented, we intended to verify the effect of meat enriched with canola oil as a source of fat, combined with the effects of the antioxidants selenium and vitamin E, on the lipid profile of older adults. However, in the treatment of meat enriched with canola oil, despite the different lipid profile of the meat between treatments, there was no significant effect on the parameters evaluated, as expected.

A possible explanation is that canola oil presents a good profile of fatty acids, with 11% omega-3 polyunsaturated fatty acids (PUFA), 53–59% MUFA, 22% omega-6 PUFAs and 7.1% saturated fatty acids (SFA) and an appropriate ratio of *n*-6/*n*-3 [48]. Atefi, Pishdad and Faghih [49] suggested replacing canola oil with sunflower oil for reducing inflammation and oxidative stress; however, they also cited other studies with canola oil’s positive effects on oxidative stress, concluding that the short period of intervention caused non-significant results.

Besides that, canola oil is obtained from the seeds of different species of the Brassica family, as *Brassica napus* is known to have high content levels of erucic acid, a fatty acid suspected of having pathogenic potential [50]. So, there is a possibility that part of the vitamin E supplement used in some meats was used to control the oxidative stress and inflammation in older adults receiving the AO treatment.

Some limitations of this study should be considered: 1. All volunteers were recruited from a unique institution which assured that all subjects received the same meals; this is a good fact, but it restricts sample size. 2. It is difficult to reference values for the outcomes studied, so the control group was used as a baseline value for comparison. 3. The experimental period [90 days] was not long enough to detect changes in the lipid profile in the longer term. 4. While we focused on biochemical parameters such as selenium, vitamin E and lipid profile, other relevant health markers (e.g., inflammatory markers, oxidative stress indicators) could be assessed to provide a more comprehensive understanding of the effects of biofortified meat. 5. Although efforts were made to standardize diets and monitor compliance, individual variations in dietary habits could have impacted the outcomes. Nevertheless, the biofortified meat impacted the concentration of these nutrients in the older adults studied.

This study has a practical application; however, further studies with other older adult groups are necessary to verify the benefits observed in our older institutionalized participants, demonstrating the importance of biofortified meat as an alternative dietary composition or supplementation strategies to optimize the beneficial effects for consumers.

## 5. Conclusions

Feeding older adults meat biofortified with selenium and vitamin E increases the concentration of this metabolite in their blood, so biofortified meat can be an important strategy for improving nutrient ingestion for this group of people. More investigations with higher experimental units and periods can be interesting for obtaining better results.

## Figures and Tables

**Table 1:** The nutritional composition of the meat (raw) that was prepared and offered to the older adults during the experiment (mean ± standard error).

	Control Meat ^1^	Biofortified Meat ^2^		
Item	C	A	O	AO
Se and Vit E, mg/100 g of meat				
Selenium	0.0046 ^b^ ± 0.004	0.0422 ^a^ ± 0.015	0.0057 ^b^ ± 0.002	0.0367 ^a^ ± 0.031
Vitamin E	0.380 ^b^ ± 0.14	0.765 ^a^ ± 0.27	0.398 ^b^ ± 0.16	0.778 ^a^ ± 0.36
Fatty acids, g/100 g of meat				
C18:2c9 t11 (CLA)	0.78 ^b^ ± 0.05	0.85 ^b^ ± 0.05	1.27 ^a^ ± 0.05	1.08 ^a^ ± 0.05
18:0 (stearic)	16.28 ± 0.29	14.92 ± 0.29	15.09 ± 0.29	15.61 ± 0.29
C18:1 c9 (oleic)	36.41 ± 0.35	37.69 ± 0.35	37.54 ± 0.35	37.82 ± 0.35
Saturated	47.04 ^a^ ± 0.49	45.55 ^a^ ± 0.49	42.87 ^b^ ± 0.49	43.58 ^b^ ± 0.49
Mono	50.24 ^b^ ± 0.48	51.46 ^b^ ± 0.48	53.99 ^a^ ± 0.48	53.53 ^a^ ± 0.48
Poly	2.72 ± 0.12	2.99 ± 0.12	3.14 ± 0.12	2.89 ± 0.12
H/h	0.55 ^a^ ± 0.01	0.53 ^a^ ± 0.01	0.46 ^b^ ± 0.01	0.47 ^b^ ± 0.01
*n*-6/*n*-3	12.51 ^a^ ± 0.52	9.22 ^a^ ± 0.52	8.27 ^b^ ± 0.52	8.09 ^b^ ± 0.52

CLA: conjugated linoleic acid (C18:2c9 t11); saturated fatty acids (C10:0; C12:0; C14:0; C15:0; C16:0; C17:0; C18:0; C20:0); Mono: monounsaturated fatty acids (C14:1c9; C16:1c9; C17:1; C18:1 c9; C18:1 c11; C18:1 c12; C18:1 c13; C18:1 c15 C18: 1 t6-t7-t8-t9; C18: 1 t10-t11-t12; C18: 1 t16; C20: 1); Poly: polyunsaturated fatty acid (C18:2c9c12; C18:2; C20:2; C20:4; C20:5 (EPA); C22:2; C22:5 (DPA) C22:6 (DHA); H/h: hypercholesterolemic (C 14:0 + C 16:0)/hypocholesterolemic (monounsaturated + polyunsaturated); *n*-6/*n*-3; ^1^ C: control meat; ^2^ A: meat biofortified with antioxidants [selenium and vitamin E]; O: meat biofortified with canola oil; AO: meat biofortified with antioxidants [selenium and vitamin E] plus canola oil. Means within a row with different superscripts differ at 5% probability.

**Table 2:** Selenium and vitamin E concentrations in the blood of older adults, basal and 90 days after starting diet biofortification (mean ± standard error).

	Treatments ^1^				Time		*p*-Value ^2^			
Item	C	A	O	AO	Basal	90 Days	OIL	ANT	O*A	Time
Selenium, µg/L	52.69 ^B^ ± 4.63	60.65 ^A^ ± 4.36	54.61 ^B^ ± 4.36	61.05 ^A^ ± 3.70	38.02 ^b^ ± 1.22	57.25 ^a^ ± 2.14	0.93	0.04	0.42	***
Vitamin E, mg/dL	7.53 ^B^ ± 0.71	10.37 ^A^ ± 0.76	8.05 ^B^ ± 0.71	10.19 ^A^ ± 0.69	10.44 ^b^ ± 0.42	9.04 ^a^ ± 0.36	0.09	0.04	0.54	**

^1^ C: control meat; A: meat biofortified with antioxidants [vitamin E and selenium]; O: meat biofortified with CLA; AO: meat biofortified with antioxidants [vitamin E and selenium] and CLA; ^2^ OIL: oil effect; ANT: antioxidant effect; O*A: interaction effect between antioxidants and oil; T: time effect. *** < 0.0001; ** < 0.001. Means within a row with different superscripts differ at 5% probability.

**Table 3:** Total cholesterol and fractions, triglycerides and glucose concentrations in the blood from older adults, basal and 90 days after starting diet biofortification.

	Treatments ^1^				Time		*p*-Value ^2^			
Item	C	A	O	AO	Basal	90 Days	O	A	O*A	Time
Cholesterol, mg/dL	175.95 ± 7.18	167.41 ± 7.58	182.37 ± 7.31	172.27 ± 6.90	189.43 ± 4.39	174.50 ± 3.62	0.76	0.49	0.92	**
HDL, mg/dL	35.61 ± 1.81	44.40 ± 1.95	42.79 ± 1.82	42.33 ± 1.78	44.79 ± 0.83	41.28 ± 0.92	0.38	0.05	0.18	**
LDL + VLDL, mg/dL	136.75 ± 5.46	137.41 ± 5.27	140.78 ± 5.38	133.37 ± 5.34	144.64 ± 4.72	133.30 ± 3.53	0.93	0.33	0.83	*
Triglycerides, mg/dL	130.10 ± 8.94	139.13 ± 8.52	135.82 ± 8.81	133.41 ± 8.64	126.16 ± 6.05	136.90 ± 6.59	0.47	0.85	0.44	*
Glucose, mg/dL	89.04 ± 4.02	89.68 ± 3.92	93.21 ± 3.96	85.51 ± 3.98	89.47 ± 2.85	85.18 ± 3.31	0.91	0.17	0.67	0.02

^1^ C: control meat; A: meat biofortified with antioxidants [vitamin E and selenium]; O: meat biofortified with CLA; AO: meat biofortified with antioxidants [vitamin E and selenium] and CLA; ^2^ O: oil effect; A: antioxidant effect; O*A: interaction effect between antioxidants and oil; T: time effect. ** < 0.001; * < 0.01.

## Data Availability

The data will be made available by request due to ethical.
